# Synthesis of some Mg/Co-Al type nano hydrotalcites and characterization

**DOI:** 10.1016/j.mex.2017.01.003

**Published:** 2017-01-30

**Authors:** Khadijeh Shekoohi, Fatemeh Sadat Hosseini, Amir Hossein Haghighi, Atefe Sahrayian

**Affiliations:** a Department of Chemistry, Darab Branch, Islamic Azad University, Darab 7481783143-196, Iran; b Young Researchers and Elite Club, Shiraz Branch, Islamic Azad University, Shiraz 71987 74731, Iran; c Department of Polymer Engineering, Shiraz Branch, Islamic Azad University, Shiraz 71987 74731, Iran

**Keywords:** Nano hydrotalcite, Layered double hydroxide, Coprecipitation, Morphology

## Abstract

Hydrotalcites are quite prevalent in nature and their importance is growing more and more because of their very wide range of potential applications and uses. Because hydrotalcite does not exist in significant quantities in nature, coprecipitation methods are the most used for prepartion of hydrotalcite. In this study: Two types of Nano hydrotalcite compounds containing one divalent (Mg-Al) and two divalent cation(Co-Mg-Al) were synthesized based on aqueous solutions of corresponding nitrates by co-precipitation method. The molar ratio influences structure and performance of hydrotalcite largely. The crystallinity and crystallite size of the hydrotalcite were observed to varying with molar of M^2+^/M^3+^ ratio. The structure and morphology of the Nano hydrotalcites were characterized by powder X-ray diffraction, scanning electron microscopy, Fourier-transformed Infrared spectroscopy and thermal gravimetric analysis. The crystallite size of the hydrotalcite was observed to increase when the Mg/Al molar ratio increases and, more significantly, when a second divalent cation (cobalt) was added.

## Graphical abstract

**Figure:**
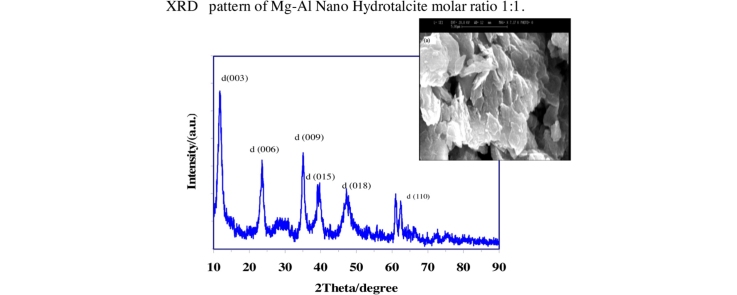

Specifications Table

**Table:** 

Subject area	*Chemistry*
More specific subject area	nanomaterial
Method name	Co-precipitation method
Name and reference of original method	Coprecipitation method is the most basic and commonly used synthetic method of nano hydrotalcite in which a mixed alkaline solution is added to a mixed salt solution and the resultant slurry is aged at a desired temperature.

## Method details

### Overview

Nano Hydrotalcites are as anionic clays or layered double hydroxides (LDH). Hydrotalcite compounds are a class of two-dimensional nanostructured anionic clays that consist of two types of metallic cations accommodated with the aid of a close packed configuration of OH groups in a positively charged brucite-like layer. The charge typically results from the substitution of the lower valence metal in the brucite structure by a metal of higher valence. The interlayer space in the hydrotalcite is, typically, occupied by water and various anions for charge Compensation [1], [2]. These compounds are often denoted as:

[M(II)_1−x_M(III)_x_(OH)_2_](A_x/n_^n−^)·*m*H_2_O

Where in, M(II) represents the divalent cations such as Mg^2+^, Fe^2+^, Ni^2+^, Cu^2+^, Co^2+^, Mn^2+^, Zn^2+^ or Cd^2+^, M(III) denotes the trivalent cations like Al^3+^, Cr^3+^, Ga^3+^ or Fe^3+^, A^n−^ is the compensating anions (CO_3_^2−^, SO_4_^2−^, Cl^−^, NO_3_^−^, organic anions), A^n−^ is an interlayer n^−^ valent anion and x varies between approximately 0.25 and 0.33 and *m* is the content of co intercalated-water [3], [4].

Synthesis methods play important role to produce nano-sized crystals of hydrotalcite, so the preparation methods are always an important research subject in this field. Many researchers have studied different synthesis methods through a lot of work on the preparation of LDHs, but mature synthesis methods include coprecipitation [5], [6], hydrothermal synthesis [7], [8], microwave radiation, ion exchange chromatography, sol-gel method [9], [10], etc. However, fibrous and extended sheet-like morphologies is reported depending upon the conditions of synthesis [11], [12].

Coprecipitation method is the most basic and commonly used synthetic method of hydrotalcite. Coprecipitation method is done in two different forms: co-precipitation at variable pH and at constant pH. In the variable pH method, a solution containing salts of the divalent and trivalent cations is added to an alkaline solution containing the anion that will be interleaved. In the coprecipitation at constant pH approach, the dissolved di and trivalent cations, the interlayer anion, and alkaline solution are all combined at the same time. The coprecipitation at constant pH requires the use of a more sophisticated experimental apparatus, but it results in particles with greater uniformity [5]. As pH is increased, the cations react with OH^−^ and (CO3)^2−^ generating hydrotalcite, which has very low solubility and therefore precipitates. These reactions are performed under strong stirring and require a further purification step to remove any remaining counter ions.

In synthesizing nanostructure hydrotalcite, some researchers suggest that through co-precipitation, separation of the nucleation and crystal growth could be done to give uniform crystallite size [13], [14], [15]. Subsequently, they used co-precipitation with high supersaturation condition, such as in colloid mill, and separate aging to synthesize the hydrotalcites. The size and shape of the nano-particles can also be controlled by pH, temperature, types of salt and their ionic strength, as well as ratio of the metal cations [16], [17], [18], [19]. Moreover, the increase in mixing rate tends to decrease the particle size [20], [21].

Currently, several types of hydrotalcites are used in various applications depending on their composition, crystallinity, thermal stability and other physicochemical properties. Some examples are catalysis, photochemistry, electrochemistry, polymerization, magnetization, biomedical science and environmental application [22], [23], [24], [25], [26], [27]. Due to their wide applications, hydrotalcites have been extensively synthesized and studied for decades. However, the structural environments and dynamical behavior of the interlayer and surface species of hydrotalcites are difficult to study and poorly understood.

A number of papers dealing with systems containing one divalent and one trivalent cation exists. A few systems containing two divalent and one trivalent cation have been reported as precursors for mixed oxide catalysts [28], [29], [30].

In this paper, binary and ternary Nano Hydrotalcites that contains magnesium, aluminum and cobalt have been synthesized by co-precipitation method at constant pH. Two types Nano Hydrotalcite with different molar ratio of Mg/Al and Mg/Al/Co were prepared and characterized from a structural point of view. Mg-Al Nano hydrotalcite with Mg/Al molar ratio of 1:1, 3:1, 5:1 and 8:1 and Co-Mg-Al Nano hydrotalcite with Co/Mg/Al molar ratio of 1:8:1 and 2:8:1 were synthesized. We study how the molar ratio influences structure and performance of Nano hydrotalcite.

## Experimental

### Material and synthesis of hydrotalcites

All materials with analytical purity are purchased from Merck and used without further purification. Four samples of magnesium-aluminum Nano hydrotalcite with varying Mg/Al ratios of 1:1, 3:1, 5:1 and 8:1 have been prepared,and two samples of cobalt- magnesium-aluminum Nano hydrotalcite with Co/Mg/Al ratios of 1:8:1, and 2:8:1 were synthesized.

The Nano hydrotalcites was synthesized by mixing of solutions A and B. For Mg-Al hydrotalcite, Solution A was prepared by mixing an equimolar solutions of Mg and Al metal nitrates 100 ml in the desired molar ratios (1:1,3:1,5:1 and 8:1). Solution B was prepared by dissolving 14 g sodium hydroxide and 10.6 g sodium carbonate in 100 ml deionised water. For Co-Mg-Al hydrotalcite,Solution A was prepared by mixing an equimolar solutions of Co,Mg and Al metal nitrates 100 ml in the desired molar ratios (1:8:1 and 2:8:1).

The mixed nitrate solution was added drop wise into solution B maintained at 45 °C. The pH was varied initially from 10 to 9 after completion of the addition of nitrates solution. Later, the precipitate was filtered and washed several times with warm distilled water to remove excess Na^+^ and NO^3−^ ions. The filtered precipitates were dried in an oven overnight at 110 °C for 10 h.

### Characterization

The synthesized Nano hydrotalcites were then characterized by X-ray diffraction (XRD), scanning electron microscope (SEM), Fourier-transformed Infrared (FTIR) spectroscopy and thermal gravimetric (TG) analysis.

X-ray diffraction (XRD) patterns were recorded using a Philips PW 1840 diffractometer under the following conditions: 40 kV, 30 mA, monochromatic CuKα radiation (λ = 0.15418 nm) over a 2θ range from 4 to 90°. Morphology was observed with a Philips XL20 scanning electron microscope. The FTIR spectra were recorded with using a Nicolet 6700 spectrometer (Thermoscientific, USA) at room temperature over a frequency range of 4000–400 cm^−1^. The samples were prepared by mixing the powdered solids with KBr. Thermogravimetric analysis was conducted under nitrogen atmosphere using Q50 TGA (TA instruments, USA) in the temperature range of 25–700 °C, with a heating rate of 10 °C/m.

## Results and discussion

### Powder X-ray diffraction

It is important to ensure that the synthesized hydrotalcites are layered structures. This was achieved using X-ray diffraction (XRD). Generally, the sharpness and intensity of XRD peak is considered to be proportional to the crystallinity. Some references are describing the relationship between crystallinity and peak intensity or sharpness [31].

**Fig. 1:**
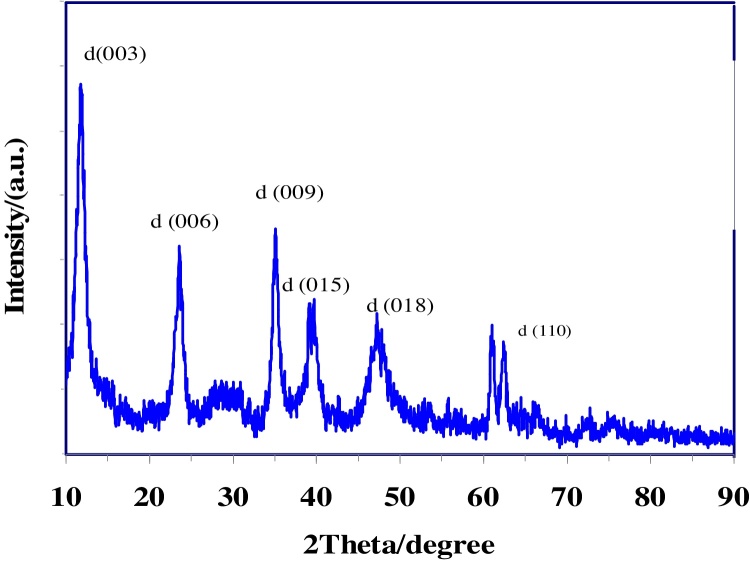
XRD pattern of Mg-Al Nano Hydrotalcite molar ratio 1:1.

**Fig. 2:**
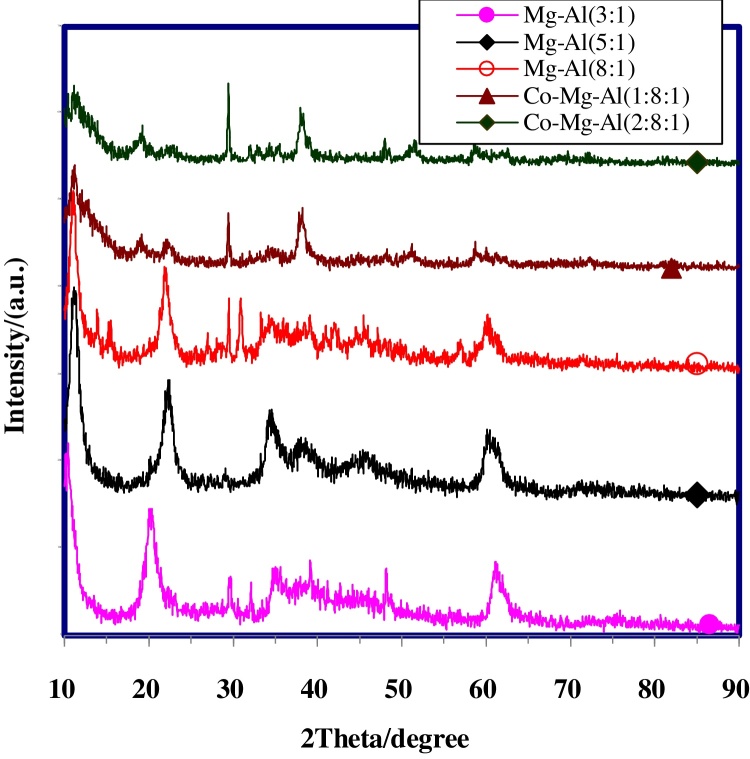
XRD pattern of Mg-Al and Co-Mg-Al Nano Hydrotalcites with different molar ratio.

The XRD pattern of Mg-Al Nano hydrotalcites with molar ratio(1:1) is represented in Fig. 1. The sharpest diffraction peak for the (003), (006), and (009) planes proves that it had the best crystallinity. Indexing of the diffraction peaks was done using standard JCPDS file. The XRD pattern of other synthesized Nano Hydrotalcites shown in Fig. 2. The difference in the intensities of the reflections indicates different degrees of crystallinity. When the cationic composition varies, these peaks became broad and their intensity decreased with increasing the molar ratio of M^2+^ to M^3+^. The XRD pattern of Co-Mg-Al Nano Hydrotalcite shown in Fig. 2, with the addition of the second divalent cation (cobalt) in hydrotalcite, the XRD d (003) peak becomes border, with increase in the moles of Co, indicating a more disordered structure or a decrease in crystallinity.

Table 1 lists the maximum diffraction intensity and the corresponding d values of the 6 samples for the (003), (006), and (009) crystal planes. The peak about at 11° 2teta for all sample of hydrotalcite are attributed to the reflections from (003) of crystallographic planes. The plane is spaced one-third per unit cell distance apart and corresponds to the interlayer.

**Table 1:** XRD patterns of synthesized Nano hydrotalcites.

Nanohydrotalcite	(003)		(006)		(009)	
	2θ (°)	d (nm)	2θ (°)	d (nm)	2θ (°)	d (nm)
Mg/Al (1:1)	11.82	0.750	23.58	0.377	35.13	0.255
Mg/Al (3:1)	10.22	0.780	20.52	0.399	35.12	0.255
Mg/Al (5:1)	11.24	0787	22.50	0.395	34.38	0.261
Mg/Al(8:1)	11.10	0.802	21.99	0.404	34.58	0.259
Co/Mg/Al(1:8:1)	10.97	0.807	22.16	0.401	34.47	0.260
Co/Mg/Al(2:8:1)	11.05	0.801	22.86	0.399	34.13	0.263

The sharp peak (003) indicates the formation of highly crystalline materials. The increase of Mg^2+^/Al^3+^ ratio causes changes in the layer spacing, as show d(003) values in Table 1. Kaneda et al. reported as the (M^2+^/M^3+^) ratio increases case the changes in the layer spacing [32].

According to the literature, the *a* and *c* values of each sample cell can be calculated based on the numerical value of the diffraction and the d value [33]. The parameter *a* of hydrotalcite corresponding to the cation-cation distance within the brucite-like layer and can be calculated by *a* = 2 × d110. On the other hand, the *c* parameter is related to the thickness of the brucite-like layer and the interlayer distance and can be obtained from *c* = 3 × d003. The calculation of unit cell parameters was done by the peaks indexing in the hexagonal crystal system is shown in Table 2. The values obtained here are close to previously reported data for similar materials [4]. The particle size can be estimated according to the Scherrer Equation:$\tau =\frac{K\lambda }{\beta cos\theta }$ (*τ* is the mean of the particle size, *K* is a dimensionless shape factor, *λ* is the X-ray wavelength, *β* is the line broadening at half the maximum intensity and – is the Bragg angle). The calculated result listed in Table 2, showing that particles size of nano hydrotalcites increases when the M^2+^/M^3+^ ratio increases. The particles size of synthesized hydrotalcite is about 43–133 nm. The Co-Mg-Al nano hydrotalcite with ratio 2:8:1 has the largest size.

**Table 2:** Lattice parameters and particle size of synthesized Nano-hydrotalcite.

Nano hydrotalcite	Lattice parameter (nm)		Particle size (nm)
	*a*	*c*	
Mg/Al (1:1)	0.3040	2.250	42.603
Mg/Al (3:1)	0.3049	2.340	50.318
Mg/Al(5:1)	0.3051	2.361	52.499
Mg/Al(8:1)	0.3063	2.406	56.796
Co/Mg/Al(1:8:1)	0.3021	2.421	86.864
Co/Mg/Al(2:8:1)	0.2984	2.403	132.895

### SEM images

In order to determine the morphology and particle size distribution of the synthesized hydrotalictes we have different respective samples which were studied by SEM.

**Fig. 3:**
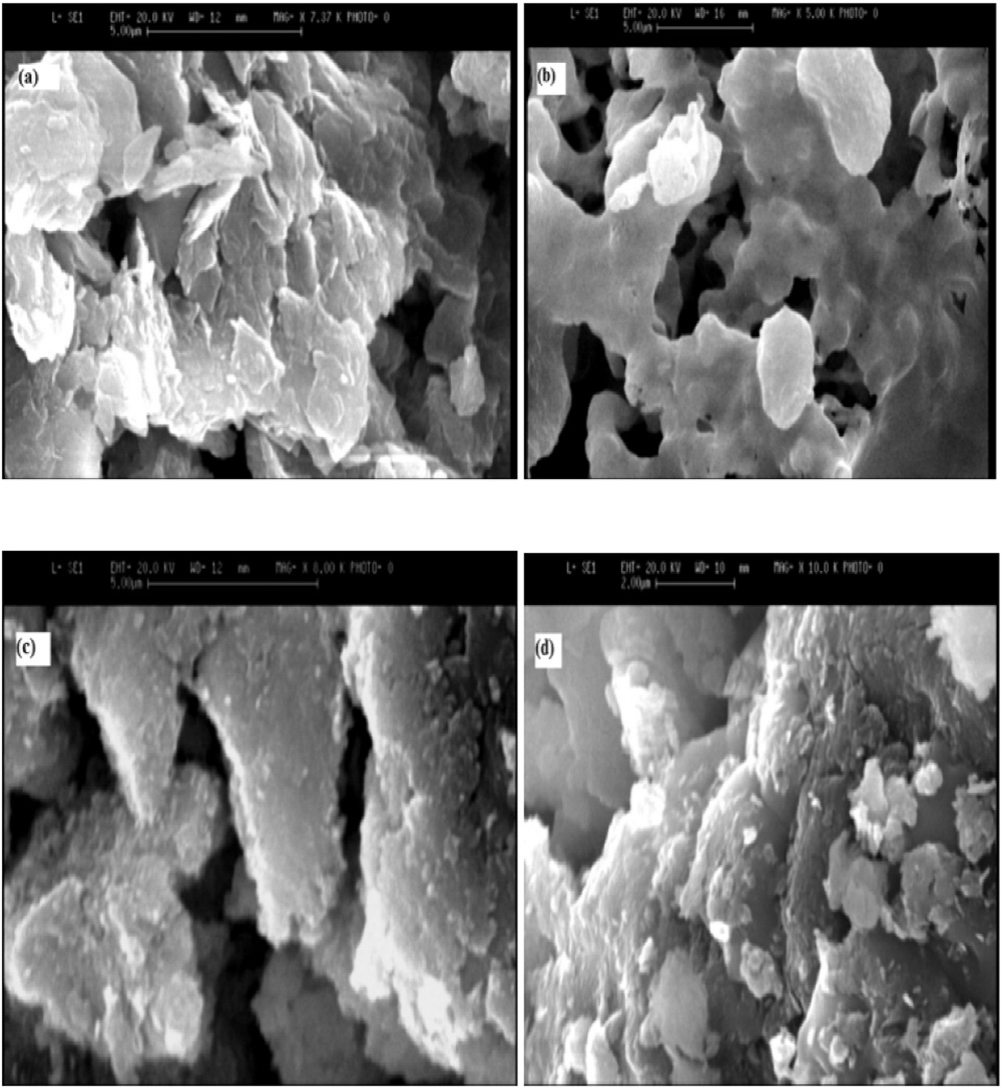
The SEM images of Nano hydrotalcites synthesized samples (Mg-Al) a- 1:1, b-8:1, (Co-Mg-Al) c- 1:8:1, d-2:8:1.

The morphologies of these samples were observed by SEM, as shown in Fig. 3. SEM images for Mg-Al Nano hydrotalcite as shown in Fig. 3a and b for Co- Mg-Al Nano hydrotalcite as shown in Fig. 3c and d. The particles were formed as an accumulation of primary nano-particles. It is observed that the as-synthesized sample has hexagonal nanosheets. When the Co content was increased the nanosheets became irregular; it was difficult to observe the hexagonal morphology, but there were still some monodisperse nanosheets.

### Fourier transform-infrared (FT-IR) spectroscopy

**Fig. 4:**
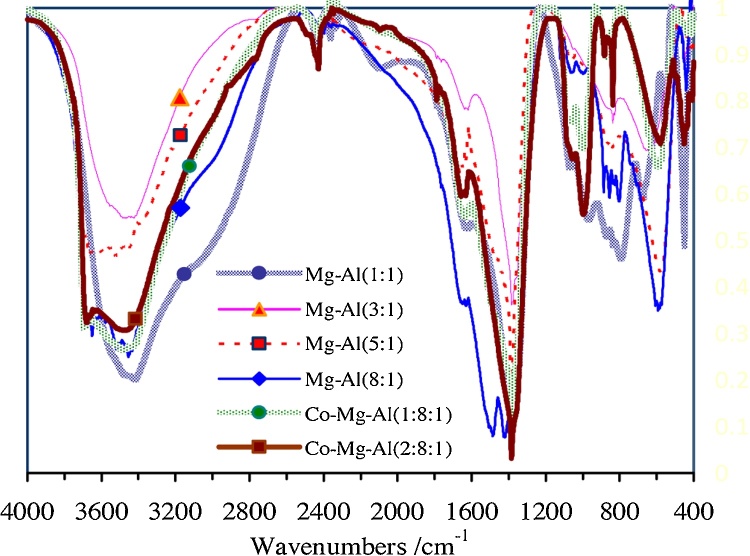
FT–IR spectra of synthesized Nano-hydrotalcite samples.

FTIR spectroscopy is another useful tool for the characterization of hydrotalcite, involving the vibrations in the octahedral lattice, the hydroxyl groups and the interlayer anions. The FTIR spectra for Mg-Al Nano hydrotalcite with molar ratio (1:1, 3:1, 5:1, 8:1) and Co-Mg-Al Nano hydrotalcite with molar ratio (1:8:1,2:8:1) in the region 400–4000 cm^−1^ are shown in Fig. 4.

The high frequency region shows a broad peak at about 3450 cm^−1^ for all samples. This broad peak due to the H-bonding stretching vibrations of the OH group is present in the brucite like sheets. Kaneda et al. reported as the (M^2+^/M^3+^) ratio increases the hydrogen stretching & bending corroborated with the changes in the layer spacing [32]. A shoulder is present around 2500 cm^−1^, is mainly attributed to hydrogen bonding between H_2_O and the anion in the interlayer. The bending vibration of the interlayer water occurs at 1600–1700 cm^−1^. The peaks at 1350–1380 cm^−1^ may indicated CO_3_^2−^ are present in the interlayer of hydrotalcite [34].

The vibrational stretching frequency of hydrogen atom in hydroxide group of Mg-Al Nano hydrotalcite with molar ratio 1:1 appears at lower wavelength 3420 cm^−1^. It has a smaller half width than the other compositions,which indicates there is a more ordered cation distribution in the former [1], [5], [18], [19].

### Thermal analysis

**Fig. 5:**
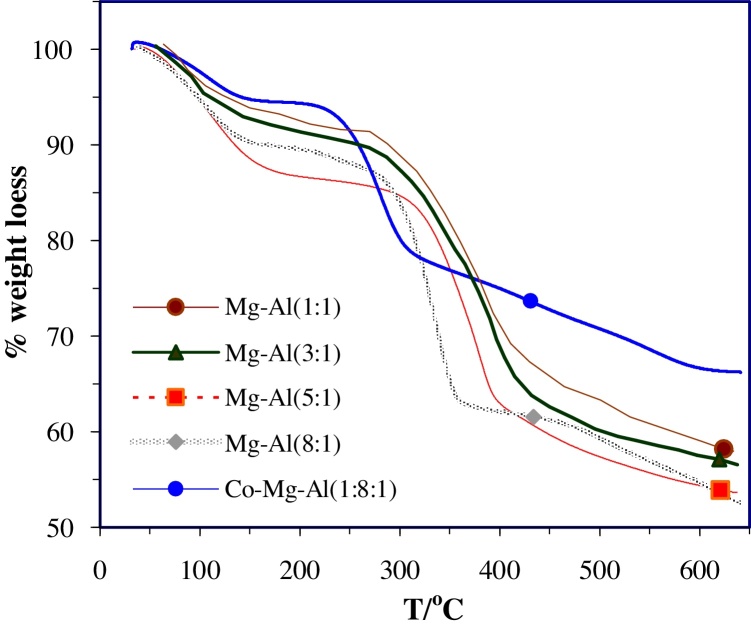
TG profile of synthesized Nano Hydrotalcites (heating rate = 10 °C/min).

To examine the thermal stability of synthesized nano hydrotalcites, TG curves are shown in Fig. 5. The TGA profiles of samples show typical two-stage decomposition profile may be divided into two well-differentiated main regions. The first weight loss occurred between 80 and 250 °C corresponding to the removal of water molecules physically adsorbed on the external surface of the crystallites. The second loss between 250 and 600 °C was ascribed to the removal of interlayer water molecules, dehydroxylation and decarbonation of the layers.

Thermogravimetric analysis indicates that synthesized Nano hydrotalcites with difference in the M^2+^/M^3+^ratio have different thermal stability. According to thermal analysis, the Mg-Al nano hydrotalcite with molar ratio 1:1 presented higher thermal stability in comparison respect to other samples. When increases M^2+^/M^3+^ratio the thermal stability become lower. With the addition of the second divalent cation (cobalt) in the brucite-like network, Co-Mg-Al nano hydrotalcite presented lower thermal stability respect to Mg-Al nano hydrotalcite as show in Fig. 5.

## Conclusion

It was confirmed that Mg-Al and Co-Mg-Al Nano hydrotalcites were successfully prepared using co-precipitation method. The chemical properties of prepared Nano hydrotalcite was affected by molar composition and showed by diffraction pattern, infrared spectra, morphology analysis, and thermal analysis as well. The XRD analysis allowed the calculation of unit cell parameters *a* and *c*. We calculated size of hydrotalcites particle using Scherrer equation. The crystallinity and crystallite size of the hydrotalcite were observed to vary with M^2+^/M^3+^ molar ratio. The increase of M^2+^/M^3+^ ratio causes changes in the layer spacing and particles sizes become larger. Co-Mg-Al nano hydrotalcites have larger particle size than Mg-Al nano hydrotalcites. Co-Mg-Al (2:8:1) have largest size respect to other samples. The crystallinity of Mg-Al hydrotalcite with ratio 1:1 was highest and that Co-Mg-Al hydrotalcites was lowest.

FTIR analysis showed that the vibration domains of interlayer anion, physically adsorbed water and vibrations of the specific bonds from hydrotalcite octahedral network were identified. Morphological analysis, by SEM method, revealed the formation of layered double hydroxides crystals, but also their cohesion, resulting in different particle sizes. According to thermal analysis, the Mg-Al nano hydrotalcite with molar ratio 1:1 presented higher thermal stability in comparison respect to other samples.

## Coprecipitation method

Coprecipitation based methods are the most used routines for preparation of nano-hydrotalcite. Coprecipitation method is done in two different forms: co-precipitation at variable pH and at constant pH. In the variable pH method, a solution containing salts of the divalent and trivalent cations is added to an alkaline solution containing the anion that will be interleaved (NaHCO3, for instance). In the coprecipitation at constant pH approach, the dissolved di and trivalent cations, the interlayer anion, and alkaline solution are all combined at the same time. The coprecipitation at constant pH requires the use of a more sophisticated experimental apparatus, but it results in particles with greater uniformity. As pH is increased, the cations react with OH^−^ and (CO3)^2−^ generating hydrotalcite, which has very low solubility and therefore precipitates. These reactions are performed under strong stirring and require a further purification step to remove any remaining counter ions.
